# High levels of population genetic differentiation in the American crocodile (*Crocodylus acutus*)

**DOI:** 10.1371/journal.pone.0235288

**Published:** 2020-07-02

**Authors:** Natalia A. Rossi, Angelica Menchaca-Rodriguez, Rafael Antelo, Byron Wilson, Kurt McLaren, Frank Mazzotti, Rafael Crespo, Joe Wasilewski, Fernando Alda, Ignacio Doadrio, Tito R. Barros, Evon Hekkala, Manuel Alonso-Tabet, Yairen Alonso-Giménez, Manuel Lopez, Georgina Espinosa-Lopez, Joe Burgess, John B. Thorbjarnarson, Joshua R. Ginsberg, Kent A. Vliet, George Amato

**Affiliations:** 1 Wildlife Conservation Society, Bronx, New York, United States of America; 2 Sackler Institute of Comparative Genomics, American Museum of Natural History, Manhattan, New York, United States of America; 3 Department of Ecology, Evolution and Environmental Biology, Columbia University, Columbia, New York, United States of America; 4 School of Biological Sciences, the University of Bristol, Bristol, England, United Kingdom; 5 Fundación Palmarito Casanare, Bogotá, Colombia; 6 Estación Biológica El Frío, Apure, Venezuela; 7 Department of Life Sciences, University of the West Indies, Mona, Jamaica; 8 Fort Lauderdale Research and Education Center, University of Florida, Fort Lauderdale, Florida, United States of America; 9 Department of Biology, Geology and Environmental Science, University of Tennessee at Chattanooga, Chattanooga, Tennessee, United States of America; 10 Departamento de Biodiversidad y Biología Evolutiva, Museo Nacional de Ciencias Naturales, Madrid, Spain; 11 Museo de Biología de la Universidad del Zulia, Maracaibo, Venezuela; 12 Department of Biological Sciences, Fordham University, Bronx, New York, United States of America; 13 Refugio de Fauna Monte Cabaniguán-Ojo de Agua, Las Tunas, Cuba; 14 Facultad de Biología, Universidad de la Habana, La Habana, Cuba; 15 US Forest Service, Idleyld Park, Oregon, United States of America; 16 Cary Institute of Ecosystem Studies, Millbrook, New York, United States of America; 17 Department of Biology, University of Florida, Gainesville, Florida, United States of America; National Cheng Kung University, TAIWAN

## Abstract

The American crocodile (*Crocodylus acutus*) is a widely distributed species across coastal and brackish areas of the Neotropical region of the Americas and the Greater Antilles. Available information on patterns of genetic differentiation in *C*. *acutus* shows a complex structuring influenced by interspecific interactions (mainly hybridization) and anthropogenic actions (mostly historical hunting, recent poaching, habitat loss and fragmentation, and unintentional translocation of individuals). In this study, we used data on mitochondrial DNA control region and 11 nuclear polymorphic microsatellite loci to assess the degree of population structure of *C*. *acutus* in South America, North America, Central America and the Greater Antilles. We used traditional genetic differentiation indices, Bayesian clustering and multivariate methods to create a more comprehensive picture of the genetic relationships within the species across its range. Analyses of mtDNA and microsatellite loci show evidence of a strong population genetic structure in the American crocodile, with unique populations in each sampling locality. Our results support previous findings showing large degrees of genetic differentiation between the continental and the Greater Antillean *C*. *acutus*. We report three new haplotypes unique to Venezuela, which are considerably less distant from the Central and North American haplotypes than to the Greater Antillean ones. Our findings reveal genetic population differentiation between Cuban and Jamaican *C*. *acutus* and offer the first evidence of strong genetic differentiation among the populations of Greater Antillean *C*. *acutus*.

## Introduction

The American crocodile (*Crocodylus acutus*) is widely distributed along coastal swamps, estuarine rivers, and lakes of the Neotropical region of the Americas and the Insular Caribbean. It is primarily a coastal crocodilian inhabiting brackish waters and the saltwater sections of rivers, coastal lagoons, and mangrove swamps [1,2]. The species also occurs in freshwater areas located inland [3–6]. This wide-ranging species overlaps with three other new world crocodilians: the Cuban crocodile (*Crocodylus rhombifer*) in Zapata, Cuba [7], Morelet’s crocodile (*Crocodylus moreletii*) in the Yucatan Peninsula, Mexico [8,9] and Belize [10], and the Orinoco crocodile *(Crocodylus intermedius)* in Venezuela [11]. It hybridizes in the wild with *C*. *moreletii* [12–14] and *C*. *rhombifer* [7,13]. The ability to tolerate and thrive in highly saline environments [15–17] allowed *C*. *acutus* to occupy a unique ecological niche and extend geographically more than any other new world crocodilian.

Like many crocodilians, populations of American crocodiles have declined due to intense hunting pressures since the early 1800s. The American crocodile was listed in the Convention on International Trade in Endangered Species of Flora and Fauna (CITES) at the Convention's inception in 1975 [18]. Since then, a combination of sustainable farming [18], habitat protection [19], and conservation and monitoring programs [20] have aided the recovery of the species. However, this recovery has not been even throughout its range, and new conservation challenges have arisen. New threats have been driven by changes in water regimes and water quality of coastal wetlands, illegal hunting [21], retaliatory killing, destruction of nests, inter- and intraspecific competition [14,22], human-mediated movements of animals across the range [23], and habitat loss and fragmentation due to coastal development [10,24]. Habitat reduction and degradation from coastal development is the leading threat to the survival of the American crocodile, as it decreases the availability of nesting sites, food resources, nursery habitat and hiding sites [8,25–27], and even contributing to increased hybridization through the breakdown of reproductive barriers and increase potential of panmixia among distinctive evolutionary lineages, leading to a decline of genetic diversity and the extinction of local populations [28]. The species is categorized as vulnerable by the International Union for Conservation of Nature (IUCN) Red List of Endangered Species [26] and listed in CITES Appendix I [19], which prohibits trade, except for the populations of Cuba [1], the Integrated Management District of Mangroves of the Bay of Cispata in Colombia, and the population of Mexico (with no export quota allowed of wild specimens for commercial purposes), all listed in Appendix II [29].

An important challenge in the conservation of any widespread species is to identify unique clusters that need to be considered as independent management units [30]. These clusters are usually defined considering habitat types within the species range, local conservation status, threats, enforcement, and legislation [19,31], as well as patterns of genetic variation within and among subpopulations. The majority of the work on *C*. *acutus* genetic variation has focused on zones where it can hybridize in the wild with *C*. *rhombifer* and *C*. *moreletti* [7,9,12,13,32,33]. Though information about genetic differentiation has been used as a tool for delineating local management units on some species of *Crocodylus* [34], there is still a need to incorporate population-level genetic information into conservation planning for many species of crocodilians, including the American crocodile.

Current information on patterns of genetic differentiation of *C*. *acutus* shows a complex population structuring influenced by interspecific interactions (mainly hybridization) and anthropogenic actions (mainly poaching, habitat destruction and fragmentation, and unintentional translocation of individuals). For example, an analysis of variation in the mtDNA control region revealed a minimum of 11 haplotypes in the American crocodile [23,32]. Nine of these haplotypes are also present in Central and North American populations [23], and only two are exclusive to the Greater Antilles [7,32,35]. *C*. *acutus* populations from South America, however, have not been incorporated in these analyses, and thus, the relationship of Antillean crocodiles with South American lineages is unclear. Moreover, hybridization with other crocodilians and human-mediated migration across the species range may affect the distribution and frequency of the reported haplotypes [23]. A study based on mitochondrial (mtDNA) and nuclear DNA markers revealed interspecific hybridization, admixture, and significant patterns of population substructure of crocodiles in Southern Florida as a result of human-mediated migrations from Latin America and the Greater Antilles [23]. More recently, a study on populations of *C*. *acutus* in the Greater Antilles and the Americas recognized distinguishable phylogenetic relationships and high genetic divergence between these two groups [36]. *C*. *acutus* populations along the Pacific coast of Costa Rica exhibited small to moderate levels of inbreeding and significant levels of population differentiation potentially attributed to the lack of connectivity between some localities and the occurrence of population bottlenecks in the past [37]. In addition, Pacheco et al [33] extensive sampling throughout Mexico revealed several unique lineages as a result of historical and present hybridization between *C*. *acutus* and *C moreletti*. In parallel to *C*. *acutus*’ species designation debates [33,36], it is imperative to strengthen the comparative research of extant populations to inform local and regional management and incorporate lacking information into the broader species assessment.

Defining conservation management units based on molecular data has proven more effective when combining a wide range of genetic markers [38–40]. The hypervariable control region of the mitochondrial DNA has been one of the most informative and widely used markers to uncover population subdivision [38,41]. In addition, nuclear polymorphic microsatellite markers have proven useful to detect subtle patterns of population structure [42–44], and to accurately assign population origin [34]. Information on population differentiation of American crocodiles derived from mtDNA markers has been useful to detect hierarchies of structuring but has not resolved subtle patterns of population subdivision [23].

To better understand patterns of genetic differentiation in the American crocodile, we used data on mtDNA and nuclear DNA to assess the degree of population structuring between and among localities in South America, North America, Central America, and the Greater Antilles. We incorporated previously unsampled areas (two sites in Jamaica, and five sites in Venezuela) and populations previously studied to create a more comprehensive picture of the genetic relationships within the species across its range. Our work aims at understanding the population genetic structuring of *C*. *acutus*, and to provide relevant information for the conservation and recovery of the species.

## Materials and methods

### Sample collection

**Fig 1:**
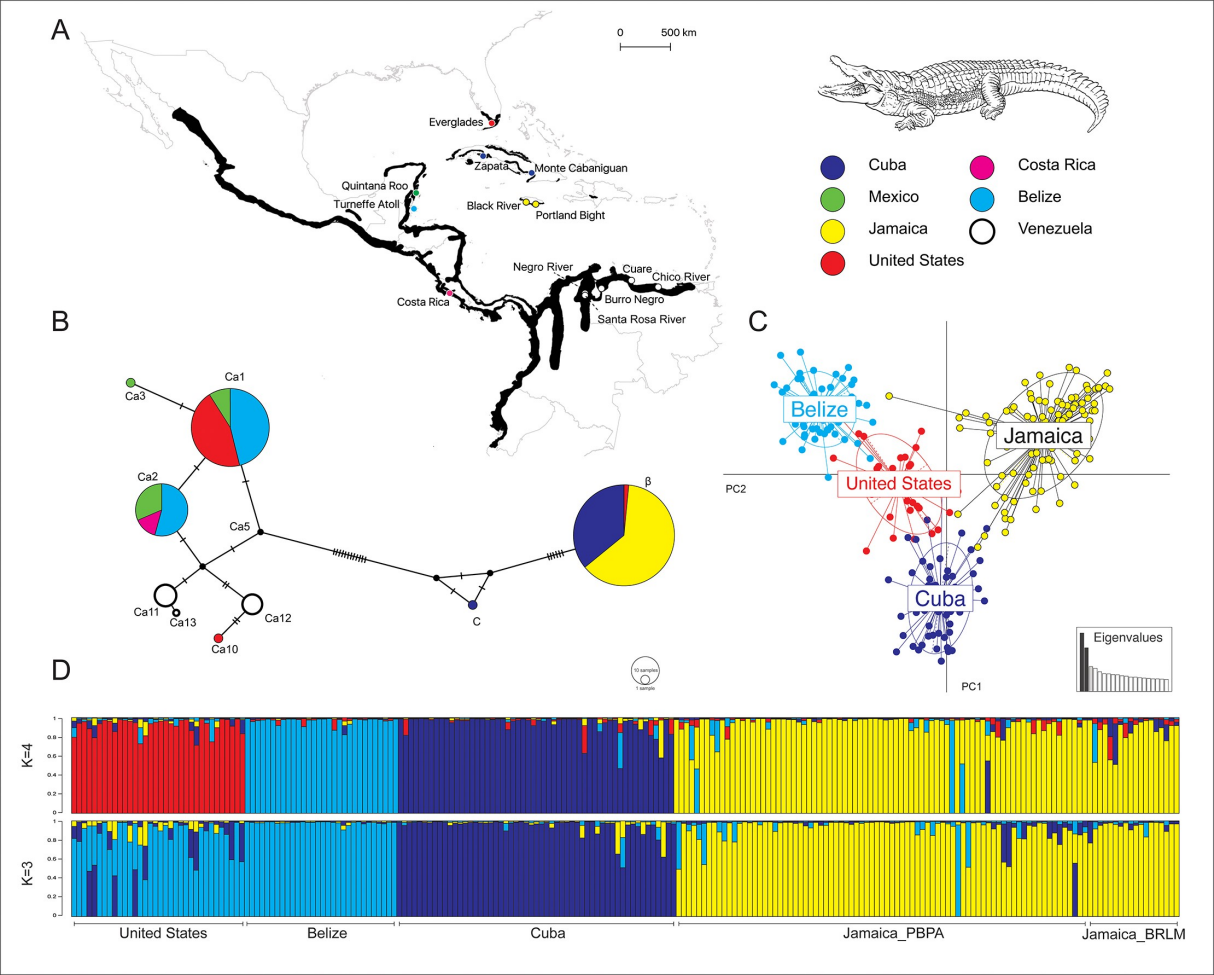
Geographic distribution, haplotype network, discriminant analysis of principal components, and population structure of the American crocodile. A) *C*. *acutus* distribution range based on most recent data of presence, and sampling localities color-coded by country (Base map credit: NASA Earth Observatory); B) Neighbor-joining haplotype network of the mtDNA control region, circle size is proportional to haplotype frequency, and hatch marks indicate mutation steps; C) scatterplot of DAPC with PC1 and PC2; D) estimated Bayesian genetic structures based on 11 microsatellite loci, with *K* = 3 and *K* = 4. Samples are colored according to geographic location.

As part of crocodilian conservation and monitoring programs, we collected tissue samples of *C*. *acutus* from five countries (United States, Belize, Venezuela, Southeastern Cuba, and Jamaica). The dataset was supplemented with mtDNA sequences obtained from Genbank from Mexico, Costa Rica, and Southwestern Cuba (Fig 1A; Table 1).

**Table 1:** Geographic distribution and description of the American crocodile samples used in this study.

Locality	Abbreviation	n	Reference	mtDNA	Microsatellites
Everglades National Park, Florida, United States	EVNP	35	this study	yes	yes
Quintana Roo, Mexico	MX	19	Ray *et al* 2004; Cedeño- Vázquez *et al* 2008	yes	no
Turneffe Atoll, Belize	BZ	31	this study	yes	yes
Costa Rica	CR	5	Rodriguez *et al*. 2011	yes	no
Chico River, Cuare, Burro Negro, Negro River, and Santa Rosa River, Venezuela	VE	11	this study	yes	no
Zapata Swamp National Park, Cuba	ZAP_CU	5	Milian-Garcia *et al*. 2015	yes	no
Wildlife Refuge Monte Cabaniguan, Cuba	WRMC_CU	60	this study	yes	yes
Black River Lower Morass, Jamaica	BRLM_JM	17	this study	yes	yes
Portland Bight Protected Area, Jamaica	PBPA_JM	82	this study	yes	yes
**Total**		**265**			

n: number of samples, mtDNA: mitochondrial DNA

Tissue samples were removed from the dorsal section of the base of the tail and stored at -20°C in 95% ethanol until DNA extraction. The sex of the individuals was unknown. Protocols for animal handling and collecting biological samples were approved by the Field Veterinary Program of the Wildlife Conservation Society and conducted following guidelines of the IUCN Crocodile Specialist Group [45]. Samples were obtained in accordance with collection permits from the National Environment and Planning Agency of Jamaica (Exemption Certificate N0 70), the Center for Inspection and Environmental Control of Cuba (License 18/15), the National Office of Biological Diversity of Venezuela (Permit number 5–0358), the Forest Department of Belize, and the Institutional Animal Care and Use Committee (IACUC) of the United States (Permits A037-2006 and D684-2007). Samples from all sites except Venezuela were transported to the Sackler Institute for Comparative Genomics at the American Museum of Natural History, New York, USA (CITES export permits C0001733 and JM2320, and USFWS permit 1018–0093) for laboratory analyses including DNA extraction, amplification and sequencing of the mtDNA control region, and amplification and sequencing of 11 microsatellite loci. Samples from Venezuela were transported to the Museo Nacional de Ciencias Naturales of Madrid, Spain (CITES permit 1395/VE9120190), where laboratory analysis, including mtDNA extraction, amplification, and sequencing were conducted. Nuclear DNA was not recovered from samples originating from Venezuela, and thus, microsatellite loci could not be included in our analyses.

### DNA extraction, sequencing and genotyping

We extracted genomic DNA from fresh scale tissue using the DNeasy Blood and Tissue kit (Qiagen, Valencia, CA) following manufacturer recommendations. The final DNA concentration of each sample was measured using a NanoDrop 2000c. A fragment of 525 base pairs (bp) of the mitochondrial control region was amplified with primers drL15459 and CR2HA [46]. Polymerase chain reaction (PCR) was performed at a final volume of 25μl with 1.25 units of AmpliTaq Gold (Applied Biosystems, Foster City, California, USA), 1x PCR Buffer, 0.2mM each dNTP, 0.2 μM each primer, 1mM MgCl_2_ and 50ng/μl DNA. An initial 2-minute denaturation cycle at 94°C was followed by 33 cycles of denaturation at 94°C for 30 seconds, annealing at 56°C for 1min, and elongation at 72°C for 7 minutes. Amplicons were purified and concentrated via ethanol precipitation. Double-stranded DNA was sequenced with the dideoxy chain termination method using an ABI 3730 automated sequencer.

To genotype samples from Belize, Cuba, Jamaica, and the Everglades, we used a panel of 11 microsatellite markers previously developed for *Crocodylus spp*., including C391, Cj16, Cj18, Cj35, Cj127, Cj119, Cj131, CUJ131, Cu5123, Cj20, Cj109 [47]. PCR reactions were prepared at a final volume of 12.5μl with 1.0 unit of AmpliTaq Gold (Applied Biosystems, Foster City, California, USA), 1x PCR Buffer, 0.4mM each dNTP, 0.1 μM fluorescently labeled forward primer, 0.1 μM reverse primer, 3.5mM MgCl_2_ and 50ng/μl DNA. Microsatellites were amplified in single PCRs for 33 cycles and three different annealing temperatures (51 ºC for Cj35 and Cj127, 62 ºC for Cj16, Cj20, and Cj109, and 58°C for all the rest). An initial 2-minute denaturation cycle at 94°C was followed by 33 cycles of denaturation at 94°C for 30 seconds, different annealing temperature for each microsatellite at 51–62°C for 1-minute, elongation at 72°C for 45 seconds and a final elongation step at 72°C for 5 minutes. We visualized PCR products using an ABI 3730 automated sequencer. Genotypes were identified using GeneMapper v5.0 software (Applied Biosystems, Foster City, California, USA). Allelic sizes were scored against the size standard GS500 LIZ.

### Haplotypic and genotypic variation

We aligned mtDNA sequences in GENEIOUS 8.1.7 (Biomatters, Ltd., San Francisco, CA, USA) using the Muscle Aligner 3.8.425 with default settings. From the 525 bp mtDNA control region fragment, we selected a 458 bp consensus region containing about 95% of the variation within our samples. We compared the mtDNA sequences obtained with those downloaded from Genbank corresponding to Mexico (AY568308-17)[48] Costa Rica (GU064561-65) [23], and Southwestern Cuba (EU034586 KM577701) [32]. We then matched sequences to previously described haplotypes [23] using DnaSP v5.10.1 software [49]. We used Rodriguez et al [23] haplotype definitions and nomenclature for our analysis. Haplotypic diversity (H_d_) [50], nucleotide diversity (π) [51,52], and the mean number of pairwise differences among sequences (K) [51–53] were calculated in Arlequin 3.5 [54,55] and DnaSP. We used TCS 1.21 software to construct statistical parsimony haplotype networks [56] in order to depict an intraspecific genealogy for our mtDNA sequences. We then built a median-joining haplotype network [57] to depict patterns of genetic variation among haplotypes using the PopART 1.7 software [58].

We used Arlequin to calculate the number of alleles, observed (H_O_) and expected heterozygosities (H_E_) in our microsatellite marker set. We used GENEPOP 4.3 [59] to test Hardy-Weinberg (HW) expectations per locus as well as genotypic linkage disequilibrium (LD) between loci. Sequential Bonferroni corrections [60] were used to adjust departures for HW and LD (p≤0.05). We took into account that genotyping errors could occur as a result of primer-site mutations or contamination and could produce null alleles and/or allele drop out. Accordingly, genotyping inconsistencies were assessed using Micro-checker 2.2.3 software [61].

### Analysis of population structure

#### Mitochondrial sequence data

The mtDNA dataset included sequences from 265 individuals corresponding to thirteen locations in seven countries (Fig 1A; Table 1): 1) Everglades National Park, the United States, 2) Quintana Roo, Mexico, 3) Turneffe Atoll, Belize, 4) Costa Rica, 5) Chico River, Cuare, Burro Negro, Negro River, and Santa Rosa River, Venezuela, 6) Zapata Swamp National Park, and the Wildlife Refuge Monte Cabaniguan, Cuba, 7) Black River Lower Morass, and Portland Bight Protected Area, Jamaica.

The partitioning of genetic variation among putative populations was assessed through a nested analysis of molecular variance (AMOVA) implemented in Arlequin. This allows for testing hypotheses of among-group and within-group differences at several hierarchical levels. We computed genetic distances in Arlequin, including pairwise F_ST_ (haplotype frequencies only) and Φ_ST_ statistics (pairwise differences between haplotypes). The significance of values (Fst and ΦST) was tested using 10,000 nonparametric random permutations. Chi-square analysis was conducted in DnaSP to test for significant differences in haplotype frequency distributions among sampling localities.

#### Microsatellite data

We analyzed microsatellite data from 225 individuals from four countries: 1) Turneffe Atoll, Belize, 2) Wildlife Refuge Monte Cabaniguan, Cuba, 3) Black River Lower Morass, and Portland Bight Protected Area, Jamaica, and 4) Everglades National Park, United States (Fig 1; Table 1, and S1 Table). We assessed spatial structure through AMOVA analysis, and estimated pairwise F_ST_ statistics in Arlequin, using Weir and Cockerham’s estimator [62], which assumes an infinite allele model of mutation. We evaluated the degree of genetic partitioning among putative populations using spatially explicit Bayesian clustering methods. Such statistical methods describe and quantify the geographic patterns of intraspecific genetic variation.

The package Geneland 3.1.4 [63,64] implemented in R 3.2.3 [65] uses an algorithm based on a spatial model which assigns individuals into a number of genetic clusters (*K*) making use of genotypes as well as spatial coordinates of sampled individuals. As is common to all explicit clustering methods, Geneland weights information of an individual's location in the search for the most likely *K* instead of assuming that all clustering solutions are equally possible. Whereas, STRUCTURE 2.3.4 [66–68] uses the number of populations (*K*) as a fixed parameter to estimate the log-likelihood of the data for the pre-defined *K* values, and assigns memberships for all individuals in the total sample set. Both programs infer unknown parameters through Markov Chain Monte-Carlo (MCMC) computations, assume Hardy-Weinberg equilibrium with linkage equilibrium between loci, and do not require an *a priori* definition of putative populations. STRUCTURE, too, allows for the incorporation of sampling location priors. Location priors allow STRUCTURE to assign individuals into genetically similar clusters considering *a priori* the geographic origin of each individual [69].

For Geneland, we determined *K* across 10 independent runs with 1,000,000 MCMC iterations and allowing *K* to vary from 1 to 8. We used a correlated allele model and set the maximum rate of the Poisson process at 225 (the number of individuals) and the maximum number of nuclei in the Poisson-Voronoi tessellation at 675 (three times the number of individuals) as suggested by Guillot et al. [63]. The uncertainty of spatial coordinates of the collection was set at 20 meters. In our study, the uncertainty of spatial coordinates accounted for the recording error. We inferred the most likely number of clusters as the modal *K* with the highest posterior probability.

We ran STRUCTURE without sampling location priors, through 20 independent runs for *K* = 1–8. We set a burn-in period of 100,000 and 1,000,000 MCMC iterations to identify the genetic clusters. We assumed an admixture model with correlated allele frequencies, which allows individuals to have mixed ancestry. We determined the optimal value of *K* according to the Δ*K* method [70] using STRUCTURE HARVESTER 0.6.94 online application [71]. We performed consensus analyses for the average scores for the inferred *K* value in CLUMPP 1.1.2 software [71].

Finally, we performed a Discriminant Analysis of Principal Components (DAPC) using the Adegenet 2.0.1 package [72] to further explore the structure of the population without making assumptions of panmixia and to complement Bayesian inference analyses. This method is useful to identify population clusters without prior knowledge and provides a robust alternative to traditional Bayesian approaches [73].

## Results

### Genetic diversity

#### Mitochondrial sequence data

We identified a total of ten mtDNA haplotypes across populations of *C*. *acutus* in the Americas and Greater Antilles (Table 2). Four haplotypes had not been previously described (GeneBank accession numbers MT416744, MT416745, MT416746, and MT416747), while the other six matched previously identified ones [23,74]. Haplotype *β*, described by Cedeño-Vazquez et al. [74], was observed in the Greater Antillean population. The most common haplotype in the Everglades was *Ca1;* noteworthy, we also found two individuals with haplotype *β* and one with a newly reported one (*Ca10*). Crocodiles from Belize exhibited the two more common haplotypes for the species in Central and North America (*Ca1* and *Ca2*). We found three haplotypes for South American crocodiles (*Ca11*, *Ca12*, and *Ca13*).

**Table 2:** Genetic diversity indices for mtDNA data.

Country	Locality	n	Haplotypes	H_d_	π	K
United States	EVNP	35	Ca1 [35], β [2], Ca10 [1]	0.15 (0.08)	0.01 (0.01)	2.36 (0.91)
Mexico	MX	19	Ca1 [7], Ca2 [10], Ca3 [1], Ca5 [1]	0.61 (0.08)	0.00 (0.00)	0.73 (0.52)
Belize	BZ	31	Ca1 [24], Ca2 [7]	0.46 (0.04)	0.00 (0.00)	0.46 (0.40)
Costa Rica	CR	5	Ca2 [5]	0.00 (0.00)	0.00 (0.00)	0.00 (0.00)
Venezuela	VE	11	Ca11 [5], Ca12 [5], Ca13 [1]	0.55 (0.07)	0.00 (0.00)	1.64 (0.81)
Cuba	ZAP_CU	5	C [1], β [4]	0.40 (0.24)	0.01 (0.00)	2.80 (1.18)
Cuba	WRMC_CU	60	β [43]	0.00 (0.00)	0.00 (0.00)	0.00 (0.00)
Jamaica	BRLM_JM	17	β [16]	0.00 (0.00)	0.00 (0.00)	0.00 (0.00)
Jamaica	PBPA_JM	82	β [66]	0.00 (0.00)	0.00 (0.00)	0.00 (0.00)
		**265**		**0.63 (0.02)**	**0.02 (0.00)**	**10.47(3.52)**

n: sample size, H(n): number of haplotypes, Hd: haplotypic diversity, π: nucleotide diversity, and K: mean number of pairwise differences among sequences. Haplotype counts are presented in brackets and standard errors in parentheses. Total number of samples and average Hd, π and K are highlighted in bold.

Crocodiles from Jamaica and one locality in Cuba (WRMC) showed the lowest values of mtDNA genetic diversity (Table 2). Maternal lineages sampled from these populations seemed to be identical in terms of molecular distances, with the same mtDNA haplotype (*β*) and values of zero for pairwise differences among sequences and nucleotide diversity. Likewise, samples from Costa Rica had values of zero in all genetic diversity estimators and only one haplotype. However, as GeneBank available sequences encompassed only five samples, this may not represent the true number of haplotypes present in Costa Rica. Samples from Mexico exhibited the highest values of haplotypic diversity, followed by samples from Venezuela, Cuba (ZAP_CU), and the Everglades in the United States. Molecular distances, however, were relatively similar among Mexican samples and were higher for Zapata, the Everglades, and Venezuela.

We detected strong patterns of haplotype distribution across geographic locations. The median-joining haplotype network (Fig 1B) reveals differences in haplotype identity between the Americas and the Greater Antilles, in accordance with recent findings [36]. Cuba and Jamaica comprise a cluster separated from all other populations. Haplotypes present in *C*. *acutus* from the Everglades, Central America, and Venezuela are closely related than those in the Antilles.

#### Microsatellite data

The analysis of microsatellite data did not find evidence of LD, and the null hypothesis of HW equilibrium could not be rejected (p > 0.05). We found no significant differences between the expected heterozygosity under HW and the observed heterozygosity in the data for any of the putative populations (Table 3). Loci Cj109 and Cj127 had the highest allele counts, while CUJ131 and CUD68 showed the lowest.

**Table 3:** Genetic diversity indices of the microsatellite data.

Country	Locality	n	Mean # alleles	H_O_	H_E_	Cj16	Cj18	Cj20	Cj35	Cj109	Cj119	Cj127	Cj131	CU5123	CUD68
United States	EVNP	35	6.091 (3.270)	0.5615 (0.2207)	0.6289 (0.1630)	7	3	6	2	10	7	13	3	7	4
Belize	BZ	31	6.364 (2.942)	0.4970 (0.2526)	0.5027 (0.2082)	9	6	7	5	4	4	9	3	5	3
Cuba	WRMC_CU	60	7.000 (3.162)	0.4939 (0.2246)	0.5339 (0.1840)	7	9	8	7	15	9	8	4	5	5
Jamaica	BRLM_JM	17	7.273 (1.954)	0.4550 (0.2398)	0.5465 (0.2131)	7	8	11	7	7	7	8	7	9	3
Jamaica	PBPA_JM	82	3.909 (1.868)	0.4545 (0.1862)	0.5446 (0.1695)	4	4	7	2	6	2	7	2	5	3
	**Total**	**225**				**34**	**30**	**39**	**23**	**42**	**29**	**45**	**19**	**31**	**18**

n: sample size, HO: observed heterozygosity, HE: expected heterozygosity. Standard deviations are in parentheses. The total number of samples and loci per locality are highlighted in bold.

The results from Micro-checker showed no evidence of scoring error due to stuttering or large allele dropout and crocodile populations from the four countries sampled (United States, Cuba, Jamaica, and Belize). There was evidence of shared null alleles (Cj20, Cj35, Cj109, Cj131) in all localities. Microsatellite null alleles are commonly encountered in population genetics studies [75] but appear to have little effect on the outcome in Bayesian assignment analyses [76].

### Population structure

#### Mitochondrial sequence data

The among-groups component of the AMOVA analysis was significant when both the haplotype frequencies and the molecular distances were considered (F_ST_: 0.96, p<0.0001; Φst = 0.75, p<0.001). Similarly, the χ^2^ showed significant differentiation among populations (χ^2^ = 659.03, p<0.001, df = 72). We found significant structure between the same pairs of populations when performing the exact test of population differentiation (Table 4). Pairwise comparisons showed significant structuring for all pairs except for Mexico—Belize, Mexico—Costa Rica, and Cuba—Jamaica. Greater Antillean *C*. *acutus* populations exhibited strong differentiation when compared with all other populations with fixation indices ranging from 0.45 to 1 (F_ST_).

**Table 4:** Pairwise estimates of FST and ΦST between sampling localities based on mtDNA.

	EVNP	MX	BZ	CR	VE	ZAP_CU	WRMC_CU	BRLM_JM	PBPA_JM
EVNP		**0.4833**	**0.2106**	**0.8689**	**0.7089**	**0.7996**	**0.9244**	**0.8891**	**0.9405**
MX	0.2775*		0.0783	0.1920	**0.3763**	**0.4531**	**0.7922**	**0.6732**	**0.841**
BZ	0.0907*	0.0397		**0.4709**	**0.4841**	**0.5527**	**0.7479**	**0.6781**	**0.7866**
CR	0.9239*	0.1667	0.4242*		**0.5845**	**0.8000**	**1.0000**	**1.0000**	**1.0000**
VE	0.6057*	0.3748*	0.4515*	0.6818*	** **	**0.4514**	**0.8519**	**0.7309**	**0.8927**
ZAP_CU	0.6818*	0.4930*	0.5697*	0.8000*	0.4818*		0.5094	0.2558	0.6179
WRMC_CU	0.8713*	0.6930*	0.7697*	1.0000*	0.6818*	0.0000		0.0000	0.0000
BRLM_JM	0.8713*	0.6930*	0.7697*	1.0000*	0.6818*	0.0000	0.0000		0.0000
PBPA_JM	0.8713*	0.6930*	0.7697*	1.0000*	0.6818*	0.0000	0.0000	0.0000	

Pairwise Fst and Φst values above and below the diagonal, respectively. Statistically significant values after sequential Bonferroni correction for pairwise comparisons with p<0.05 are highlighted in bold. Asterisks (*) below the diagonal indicate significant values (p<0.05) for the exact test of population differentiation.

#### Microsatellite data

The among-groups component of the AMOVA analysis was significant (F_ST_: 0.20, p<0.0001). In contrast to the data from the mtDNA, pairwise comparisons between Greater Antillean populations from Cuba and Jamaica did reveal population structure; congruent to the mtDNA analysis, all remaining comparisons between population pairs were also significant (Table 5).

**Table 5:** Pairwise estimates of FST between sampling localities based on mtDNA.

	EVNP	BZ	WRMC_CU	BRLM_JM	PBPA_JM
EVNP	-	**0.1240**	**0.2163**	**0.1733**	**0.1797**
BZ		-	**0.3035**	**0.2704**	**0.3214**
WRMC_CU			-	**0.2363**	**0.1251**
BRLM_JM				-	0.0195
PBPA_JM					-

Statistically significant values for pairwise comparisons with p<0.05 are highlighted in bold.

Spatially explicit Bayesian clustering analysis implemented in Geneland yielded a modal number of four populations. All ten independent runs converged in four populations. The comparison of the posterior probability of assignment of individuals to populations allocated each individual to the population from which it originated. Non-spatial Bayesian clustering implemented in STRUCTURE with no prior distribution revealed subdivision of the samples into three populations (*K* = 3) with Delta *K* = 950.33 (Fig 1D); clustering individuals from the United States and Belize and separating Cuba from Jamaica. The second-best configuration assigned individuals into four populations: Belize, Cuba, Jamaica, and the United States with Delta *K* = 147.37 (Fig 1D).

The DACP (Fig 1C) suggested four distinct groups: (1) Belize, (2) Cuba (3) Jamaica, and (4) the United States. This grouping was based predominantly on the first principal component (PC1), which explained 19.23% of the variance in allele frequencies among samples and was augmented by PC2 (12.50%) and, to a lesser extent by PC3 (5.76%).

## Discussion

We present new and relevant information to understanding *C*. *acutus* evolutionary history and reveal patterns of genetic partitioning important for its conservation. As a widespread species living across a diversity of fresh and brackish water habitats, able to move long distances across saltwater, the American crocodile has been considered "homogenous" across its range before population studies emerged in the early 2000s. With most research focusing on hybrid zones [7,9,19,32,74], our study contributes to current debates on the taxonomic complexity of *C*. *acutus* [33,35,36]. Overall, our findings improve our understanding of populations of *C*. *acutus* across seven countries and provide sound evidence of genetic structuring with direct consequences into the management of local, unique populations.

Analyses for both mtDNA and nuclear markers show evidence of population genetic structure in the American crocodile with unique populations in North America, Central America, South America, and the Greater Antilles. In accord with other studies [7,23,35,36], our results show the greatest degree of genetic differentiation between the continental and Greater Antillean *C*. *acutus*. The suite of genetic differentiation estimators used in our mtDNA analysis indicated that this differentiation is strong, reaching pairwise comparisons estimator values (F_ST_ and Φ_ST_) as high as one between Greater Antillean and continental populations. The same estimators found a lack of differentiation between populations in Mexico and Costa Rica, suggesting deep matrilineal phylogenetic divergences between Central America populations and their conspecifics in the Insular Caribbean.

We report four newly discovered haplotypes: three in Venezuela (*Ca11*, *Ca12*, and *Ca13*) and one in the Everglades (*Ca10*). Our results show that Venezuelan haplotypes are considerably less distant from Central and North American haplotypes than Greater Antillean haplotypes (Fig 1B). Notably, the addition of South American sampling locations into haplotype designation was useful to improve the understanding of *C*. *acutus* intraspecific population genealogies.

A study by Bloor et al. [77], using mtDNA cytochrome b and cytochrome oxidase I gene sequences in captive *C*. *acutus* from Colombia, revealed two distinct lineages: one closely related to Central American haplotypes and a second one unique to Colombian *C*. *acutus*. Our findings strengthen such evidence and highlight the importance of future research to better understand haplotype relationships within South American *C*. *acutus*.

In addition, all but one sample from the Greater Antilles share the same haplotype (*β*). Haplotype *β* is closer to Cuban crocodile haplotype *α* than to any other haplotypes from the Americas [7,32,36]; mitochondrial capture may have occurred during an ancient hybridization event between Greater Antillean *C*. *acutus* and *C*. *rhombifer* [23]. An alternative scenario postulates multiple colonization events to Cuba by *C*. *rhombifer* and later by *C*. *acutus*, with sustained periods of hybridization and dispersion across the island [32]. In fact, hybridization between Cuban and American crocodiles in Cuba seems to have taken place both historically and in recent times [7]. Similarly, ancestral and present hybridization has occurred between the American and Morelet’s crocodiles in the Yucatan Peninsula [9,33]. Regardless of potential evolutionary explanations for haplotype origins, the geographic distribution of *C*. *acutus* haplotypes suggests that strong genetic structuring shaped haplotype identities, followed by differences in haplotype frequencies.

The new haplotype (*Ca10*) found in the Everglades seems to be closer to South and Central American haplotypes. In addition, we found two individuals in the Everglades with haplotype *β*. Rodriguez et al. [23] reported the presence of several haplotypes from Latin America and the Caribbean within Southern Florida and attributed genetic structuring as a result of the admixture of local haplotypes with those of foreign and captive American crocodiles. In addition, the authors reported only one haplotype (*Ca1*) in core nesting areas in the Everglades. In this context, the two additional haplotypes present in our samples from the Everglades (*β* and *Ca10*) are most likely introduced or admixed individuals, suggesting that individuals with foreign haplotypes are being released and/or moved from areas nearby, where non-local haplotypes have been previously reported.

Our analysis of microsatellite data further uncovered patterns of genetic subdivision between populations of the Greater Antilles, Belize, and the United States with at least three populations. It is more likely that four populations occur within these sampling locations, as found by Geneland and the DAPC, as the Evanno method may underestimate *K* when a hierarchical structure is present. Spatial explicit models applied to other studies have detected biologically meaningful clusters where STRUCTURE failed to detect any population subdivision [78,79] and might be more accurate for populations exhibiting some degree of isolation by distance [80]. Overall, we found sound evidence supporting that Cuban and Jamaican *C*. *acutus* represent two distinct populations. The presence of admixture in Jamaica and Cuba could be explained by natural or human-mediated migration of individuals between localities.

## Conclusion

Although sampling was limited and extremely challenging as a result of the current political and administrative environment where *C*. *acutus* is distributed, our study provides a thorough comparison among populations across the species range and robust evidence of genetic differentiation among populations of the Greater Antilles. With this evidence and that of previous studies [36], we propose the incorporation of independent conservation management units for Cuban and Jamaican *C*. *acutus*. Our uneven allocation of sampling effort towards Portland Bright Protected Area versus the area around Black River Lower Morass prevented a more even sample across localities in Jamaica. We strongly suggest that future studies expand the sampling area to further clarify potential patterns of subtle structuring between American crocodiles at these two locations in Jamaica.

Information at the regional-scale is crucial for regional planning and conservation of the species. Importantly, in the context of increasing coastal development throughout the range of the American crocodile, with habitat loss and fragmentation jeopardizing local populations [24,81,82], having readily-available genetic information at the population level will be critical to inform country and site-specific management plans, aid *ex-situ* conservation efforts, and support the implementation of reintroduction programs.

Future research should also incorporate comparative analysis for other sampling localities in South America and Central America. A broader assessment of *C*. *acutus* will further aid conservation efforts and population management decisions to achieve an effective range-wide conservation strategy for the species. Additionally, future studies should aim at using whole-genome sequencing to resolve population structuring further and to understand the phylogenetic evolution of the American crocodile and its relationships with other crocodilian species in the Americas and the Insular Caribbean. Finally, as research looks deeper into *C*. *acutus* lineages across its range and suggests the occurrence of potential cryptic species as a result of ancient hybridization and other evolutionary processes [33,36], our study aims at providing population-level data to inform current management and conservation as we continue our debate into *C*. *acutus* species designations.

## Supporting information

S1 Table Table of microsatellite genotypes for *C*. *acutus* samples collected in this study. (XLS)
