# Supplementary material for: High levels of population genetic differentiation in the American crocodile (Crocodylus acutus)
Source: PLoS One. 2020 Jul 2;15(7):e0235288. doi: 10.1371/journal.pone.0235288 (PMC7332040; doi:10.1371/journal.pone.0235288)
Supplement: Attachment — Submitted filename: Response to Reviewers.pdf [file pone.0235288.s002.pdf]

Dear academic editor and reviewers,

Thank you very much for reviewing our manuscript – your suggestions were very helpful!

Please find below our responses to each point raised in your review. We remain ready to address any additional questions or suggestions to improve our manuscript.

1. Please ensure that your manuscript meets PLOS ONE's style requirements, including those for file naming.

Thanks for pointing this styling issue. We have revised our manuscript and all additional documentation, and secure their adherence to PLOS ONE style requirements.

2. In your Methods section, please provide additional information regarding the permits you obtained for the work.

We have expanded our data collection paragraph in our Methods section of the manuscript adding the additional information requested (lines 1743-180).

3. We note that you have stated that you will provide repository information for your data at acceptance. Should your manuscript be accepted for publication, we will hold it until you provide the relevant accession numbers or DOIs necessary to access your data. If you wish to make changes to your Data Availability statement, please describe these changes in your cover letter and we will update your Data Availability statement to reflect the information you provide. (accession numbers requested in GeneBank).

We have added GeneBank accession numbers for new haplotypes described in this manuscript (lines 340-341). Microsatellite data remains available as supplementary information of the manuscript (S1).

4. Inclusion of statement in Cover Letter in regards to Competing Interests (“"This does not alter our adherence to PLOS ONE policies on sharing data and materials.”)

We have revised our original cover letter to add the above-mentioned statement.

5. Please ensure that you refer to Figure 1 in your text as, if accepted, production will need this reference to link the reader to the figure.

Thank you very much for pointing this out. We noticed that the previous document marked an error message where the tables and figures were cited. We have now corrected this problem in all relevant sections of the manuscript.

6. We note that Figure 1 in your submission contains map images which may be copyrighted.

We have replaced map images in Figure 1 in compliance with the CC BY 4.0 license. The world basemap was downloaded from NASA Earth Observatory and the species distribution

range of *C. acutus* is an original image generated for this manuscript by one of the co-authors (Rafael Antelo)

7. We note you have included a table to which you do not refer in the text of your manuscript.

Thank you very much for pointing this out. We noticed that the previous document marked an error message where the tables and figures were cited. We have now corrected this problem in all relevant sections of the manuscript.

Reviewer #1:

Many thanks for your suggestions! We have addressed each one of your comments to improve this manuscript. We agree that there are additional threats that affect *C. acutus* across its range so we have expanded a description of such in our introduction. We have also updated the bibliography and included the most recent papers that are most relevant to the genetics of the conservation of *C. acutus*. Where relevant, we have also included papers relevant to new surveys and ecological studies.

1. Author do not consider and include other threats as habitat destruction for many purposes even with more important.

We have added more background in key threats to *Crocodylus acutus*, incorporated references, and discussed the relevance of genetics in the context of habitat loss and fragmentation. Specifically, we have included a paragraph addressing the consequences of habitat destruction and other contemporary threats that could threaten the species' genetic integrity. These updates can be found in lines 76-85, 545-550, and the References section.

2.An update of bibliography is required.There are updated information about C. acutus surveys and general information and should be included.

To address this comment we have updated our References section to include those papers that better inform our introduction, discussion, and conclusion sections (see list of new references below and the updated References section in the manuscript). If you have any specific suggestions on literature we would still need to add please kindly let us know.

1. UNEP (2020). The Species+ Website. Nairobi, Kenya. Compiled by UNEP-WCMC, Cambridge, UK. Available at: www.speciesplus.net. [Accessed (04/29/2020)].

2. LeBuff C. Historical review of American crocodiles (*Crocodylus acutus*) along the Florid Gulf Coast. History. 2016;54(3):50–7.

3. IUCN CSG Crocodilian Capacity Building Manual. Available at: http://www.iucncsg.org/pages/Crocodilian-Capacity-Building-Manual-Home.html.

4. Todesco M, Pascual MA, Owens GL, Ostevik KL, Moyers BT, Hübner S, et al. Hybridization and extinction. Evol Appl. 2016;9(7):892–908.

5. Black J. Assessment of crocodile abundance and seasonal effects of salinity on distribution using both boat based and aerial drone surveys. Purdue University Graduate School; 2019.

6. Cissell JR, Steinberg MK. Human Landscape Modification in Placencia, Stann Creek District, Belize: Possible Implications for Crocodile Hybridization. J Lat Am Geogr. 2020;19(2):218–42.

7. Ortega-León AM, Santos-Morales AH, Zamora-Abrego JG, Pérez-Mendoza HA. Analysis of the population dynamics of the endangered American crocodile, *Crocodylus acutus*in Paramillo National Natural Park. Mar Freshw Res [Internet]. 2020; Available from: https://doi.org/10.1071/MF19026

8. Machkour-M'Rabet S, Hénaut Y, Charruau P, Gevrey M, Winterton P, Legal L. Between introgression events and fragmentation, islands are the last refuge for the American crocodile in Caribbean Mexico. Mar Biol. 2009;156(6):1321–33.

9. Tellez M, Boucher M. The lessons of history and the future of American Crocodile conservation in Belize. Herpetol Rev. 2018;49(3):492–8.

10. Cox K, Brumund B. Crocodile Smiles: How listed species cope in an age of urbanization. Nat Resour Environ. 2018;32(3):44–7.

11. Weaver JP, Rodriguez D, Venegas‐Anaya M, Cedeño‐Vázquez JR, Forstner MRJ, Densmore III LD. Genetic characterization of captive Cuban crocodiles (*Crocodylus rhombifer*) and evidence of hybridization with the American crocodile (*Crocodylus acutus*). J Exp Zool Part A Ecol Genet Physiol. 2008;309(10):649–60.

12. García-Grajales J, Buenrostro-Silva A. Assessment of human–crocodile conflict in Mexico: patterns, trends and hotspots areas. Mar Freshw Res. 2019;70(5):708–20.

13. Mable BK. Conservation of adaptive potential and functional diversity: integrating old and new approaches. Conserv Genet. 2019;20(1):89–100.

14. Milián-García Y, Castellanos-Labarcena J, Russello MA, Amato G. Mitogenomic investigation reveals a cryptic lineage of *Crocodylus*in Cuba. Bull Mar Sci. 2018;94(2):329–43.

15. Fitzgerald LA, Walkup D, Chyn K, Buchholtz E, Angeli N, Parker M. The future for reptiles: advances and challenges in the Anthropocene. Encycl Anthr DellaSala, D, M Goldstein (Eds) Elsevier Sci Ltd, Oxford, UK. 2018;163–74.

16. Somaweera R, Brien ML, Platt SG, Manolis C, Webber BL. Direct and indirect interactions with vegetation shape crocodylian ecology at multiple scales. Freshw Biol. 2019;64(2):257–68.

3. An update of CITES appendices for some countries should be added.

We have updated CITES listings (lines 87-90).

Reviewer #2:

Many thanks for your suggestions on highlighting the relevance of our study to inform conservation! This study will hopefully be useful to inform management plans of *C. acutus* where translocations and reintroductions could happen and where rapid habitat loss and fragmentation can put distinct populations at risk. We have expanded our discussion and introduction sections to better explain how the genetic integrity of *C. acutus* could be at risk and how our study could prevent further loss of genetic diversity. Additionally, we have further explained the purpose of the statistical analyses conducted for the mtDNA and microsatellite data.

1.It is better to discuss more how the results would improve the conservation of the American crocodile.

It is certainly important that our findings can inform conservation. We had added a paragraph in the discussion section (lines 544-550) highlighting the importance of informing future management plans to maintain the genetic integrity of the populations.

2.It would be helpful for the non-specialist reader if the authors provide the general-purpose and characteristic of each statistical analysis.

To further describe the characteristics and purpose of the statistical analysis conducted in our study, we have added lines (299-300) for the nested AMOVA used in the mitochondrial DNA analysis; and lines (316-317) for the Bayesian clustering analyses used in the microsatellite study. In addition, in lines 289-299, 319-330, 334-336, and 356-361 we explained more in-depth the statistical analyses performed.
