# Supplementary material for: High levels of population genetic differentiation in the American crocodile (Crocodylus acutus)
Source: PLoS One. 2020 Jul 2;15(7):e0235288. doi: 10.1371/journal.pone.0235288 (PMC7332040; doi:10.1371/journal.pone.0235288)
Supplement: Peer review history

# Peer review: Decision Letter 0

**DOI:** 10.1371/journal.pone.0235288.r001

**Authors:** Tzen-Yuh Chiang

29 Apr 2020

PONE-D-20-06980

High levels of population genetic differentiation in the American crocodile (Crocodylus acutus)

PLOS ONE

Dear Dr. Rossi,

Thank you for submitting your manuscript to PLOS ONE. After careful consideration, we feel that it has merit but does not fully meet PLOS ONE’s publication criteria as it currently stands. Therefore, we invite you to submit a revised version of the manuscript that addresses the points raised during the review process.

We would appreciate receiving your revised manuscript by Jun 13 2020 11:59PM. When you are ready to submit your revision, log on to https://www.editorialmanager.com/pone/ and select the 'Submissions Needing Revision' folder to locate your manuscript file.

If you would like to make changes to your financial disclosure, please include your updated statement in your cover letter.

To enhance the reproducibility of your results, we recommend that if applicable you deposit your laboratory protocols in protocols.io, where a protocol can be assigned its own identifier (DOI) such that it can be cited independently in the future. For instructions see: http://journals.plos.org/plosone/s/submission-guidelines#loc-laboratory-protocols

Please include the following items when submitting your revised manuscript:

- A rebuttal letter that responds to each point raised by the academic editor and reviewer(s). This letter should be uploaded as separate file and labeled 'Response to Reviewers'.
- A marked-up copy of your manuscript that highlights changes made to the original version. This file should be uploaded as separate file and labeled 'Revised Manuscript with Track Changes'.
- An unmarked version of your revised paper without tracked changes. This file should be uploaded as separate file and labeled 'Manuscript'.

Please note while forming your response, if your article is accepted, you may have the opportunity to make the peer review history publicly available. The record will include editor decision letters (with reviews) and your responses to reviewer comments. If eligible, we will contact you to opt in or out.

We look forward to receiving your revised manuscript.

Kind regards,

Tzen-Yuh Chiang

Academic Editor

PLOS ONE

Journal requirements:

When submitting your revision, we need you to address these additional requirements:

1.    Please ensure that your manuscript meets PLOS ONE's style requirements, including those for file naming. The PLOS ONE style templates can be found at http://www.plosone.org/attachments/PLOSOne_formatting_sample_main_body.pdf and http://www.plosone.org/attachments/PLOSOne_formatting_sample_title_authors_affiliations.pdf

2. In your Methods section, please provide additional information regarding the permits you obtained for the work. Please ensure you have included the full name of the local authority that approved collection of samples in each territory, if no permits were required, a brief statement explaining why.

3. We note that you have stated that you will provide repository information for your data at acceptance. Should your manuscript be accepted for publication, we will hold it until you provide the relevant accession numbers or DOIs necessary to access your data. If you wish to make changes to your Data Availability statement, please describe these changes in your cover letter and we will update your Data Availability statement to reflect the information you provide.

4. Thank you for stating the following in the Competing Interests section:

"Dr. Rossi’s research was carried out with financial support of the Sackler Institute of Comparative Genomics of the American Museum of Natural History (AMNH) (https://www.amnh.org/research/sicg ). AMNH support included the provisioning of laboratory space, laboratory materials and technical training. The Wildlife Conservation Society (https://www.wcs.org/) provided financial support to carry out C. acutus ‘skin tissue sample collection in the Wildlife Refuge Monte Cabaniguan, Cuba. The Department of Ecology, Evolution and Environmental Biology (http://e3b.columbia.edu/ ) and the Institute of Latin American Studies at Columbia University (http://ilas.columbia.edu/ ) in the City of New York provided financial support via travel grants for Dr. Rossi to conduct fieldwork at the Wildlife Refuge Monte Cabaniguan in Cuba and the Everglades National Park in the United States."

Please confirm that this does not alter your adherence to all PLOS ONE policies on sharing data and materials, by including the following statement: "This does not alter our adherence to  PLOS ONE policies on sharing data and materials.” (as detailed online in our guide for authors http://journals.plos.org/plosone/s/competing-interests).  If there are restrictions on sharing of data and/or materials, please state these. Please note that we cannot proceed with consideration of your article until this information has been declared.

Please include your updated Competing Interests statement in your cover letter; we will change the online submission form on your behalf.

Please know it is PLOS ONE policy for corresponding authors to declare, on behalf of all authors, all potential competing interests for the purposes of transparency. PLOS defines a competing interest as anything that interferes with, or could reasonably be perceived as interfering with, the full and objective presentation, peer review, editorial decision-making, or publication of research or non-research articles submitted to one of the journals. Competing interests can be financial or non-financial, professional, or personal. Competing interests can arise in relationship to an organization or another person. Please follow this link to our website for more details on competing interests: http://journals.plos.org/plosone/s/competing-interests

5. Please ensure that you refer to Figure 1 in your text as, if accepted, production will need this reference to link the reader to the figure.

6. We note thatt Figure 1  in your submission contains map images which may be copyrighted. All PLOS content is published under the Creative Commons Attribution License (CC BY 4.0), which means that the manuscript, images, and Supporting Information files will be freely available online, and any third party is permitted to access, download, copy, distribute, and use these materials in any way, even commercially, with proper attribution. For these reasons, we cannot publish previously copyrighted maps or satellite images created using proprietary data, such as Google software (Google Maps, Street View, and Earth). For more information, see our copyright guidelines: http://journals.plos.org/plosone/s/licenses-and-copyright.

We require you to either (1) present written permission from the copyright holder to publish these figures specifically under the CC BY 4.0 license, or (2) remove the figures from your submission:

1.    You may seek permission from the original copyright holder of Figure 1 to publish the content specifically under the CC BY 4.0 license. 

We recommend that you contact the original copyright holder with the Content Permission Form (http://journals.plos.org/plosone/s/file?id=7c09/content-permission-form.pdf) and the following text:

“I request permission for the open-access journal PLOS ONE to publish XXX under the Creative Commons Attribution License (CCAL) CC BY 4.0 (http://creativecommons.org/licenses/by/4.0/). Please be aware that this license allows unrestricted use and distribution, even commercially, by third parties. Please reply and provide explicit written permission to publish XXX under a CC BY license and complete the attached form.”

Please upload the completed Content Permission Form or other proof of granted permissions as an "Other" file with your submission.

In the figure caption of the copyrighted figure, please include the following text: “Reprinted from [ref] under a CC BY license, with permission from [name of publisher], original copyright [original copyright year].”

2.    If you are unable to obtain permission from the original copyright holder to publish these figures under the CC BY 4.0 license or if the copyright holder’s requirements are incompatible with the CC BY 4.0 license, please either i) remove the figure or ii) supply a replacement figure that complies with the CC BY 4.0 license. Please check copyright information on all replacement figures and update the figure caption with source information. If applicable, please specify in the figure caption text when a figure is similar but not identical to the original image and is therefore for illustrative purposes only.

The following resources for replacing copyrighted map figures may be helpful:

USGS National Map Viewer (public domain): http://viewer.nationalmap.gov/viewer/

The Gateway to Astronaut Photography of Earth (public domain): http://eol.jsc.nasa.gov/sseop/clickmap/

Maps at the CIA (public domain): https://www.cia.gov/library/publications/the-world-factbook/index.html and https://www.cia.gov/library/publications/cia-maps-publications/index.html

NASA Earth Observatory (public domain): http://earthobservatory.nasa.gov/

Landsat: http://landsat.visibleearth.nasa.gov/

USGS EROS (Earth Resources Observatory and Science (EROS) Center) (public domain): http://eros.usgs.gov/#

Natural Earth (public domain): http://www.naturalearthdata.com/

 

7. We note you have included a table to which you do not refer in the text of your manuscript. Please ensure that you refer to Table 1, 2 and 5 in your text; if accepted, production will need this reference to link the reader to the Table.

[Note: HTML markup is below. Please do not edit.]

Reviewers' comments:

Reviewer's Responses to Questions

**Comments to the Author**

1. Is the manuscript technically sound, and do the data support the conclusions?

The manuscript must describe a technically sound piece of scientific research with data that supports the conclusions. Experiments must have been conducted rigorously, with appropriate controls, replication, and sample sizes. The conclusions must be drawn appropriately based on the data presented. 

Reviewer #1: Yes

Reviewer #2: Yes

**********

2. Has the statistical analysis been performed appropriately and rigorously? 

Reviewer #1: Yes

Reviewer #2: Yes

**********

3. Have the authors made all data underlying the findings in their manuscript fully available?

The PLOS Data policy requires authors to make all data underlying the findings described in their manuscript fully available without restriction, with rare exception (please refer to the Data Availability Statement in the manuscript PDF file). The data should be provided as part of the manuscript or its supporting information, or deposited to a public repository. For example, in addition to summary statistics, the data points behind means, medians and variance measures should be available. If there are restrictions on publicly sharing data—e.g. participant privacy or use of data from a third party—those must be specified.

Reviewer #1: Yes

Reviewer #2: Yes

**********

4. Is the manuscript presented in an intelligible fashion and written in standard English?

PLOS ONE does not copyedit accepted manuscripts, so the language in submitted articles must be clear, correct, and unambiguous. Any typographical or grammatical errors should be corrected at revision, so please note any specific errors here.

Reviewer #1: Yes

Reviewer #2: Yes

**********

5. Review Comments to the Author

Please use the space provided to explain your answers to the questions above. You may also include additional comments for the author, including concerns about dual publication, research ethics, or publication ethics. (Please upload your review as an attachment if it exceeds 20,000 characters)

Reviewer #1: Author do not consider and include other threats as habitat destruction for many purposes even with more important.

An update of bibliography is required. There are updated information about C. acutus surveys and general information and should be included.

Also, an update of CITES app for some countries should be added.

Reviewer #2: The authors addressed an exciting and essential question, which was population genetics of the American crocodile, to provide relevant information for the conservation and recovery of the species. Results revealed genetic differentiation between the Cuban and Jamaican populations and among the Greater Antillean populations.

I enjoyed reading this manuscript. It is well-written and organized other than errors on links to figure 1. It is better to discuss more how the results would improve the conservation of the American crocodile. It would be helpful for the non-specialist reader (like me) if the authors provide the general-purpose and characteristic of each statistical analysis.

**********

6. PLOS authors have the option to publish the peer review history of their article (what does this mean?). If published, this will include your full peer review and any attached files.

If you choose “no”, your identity will remain anonymous but your review may still be made public.

**Do you want your identity to be public for this peer review?** For information about this choice, including consent withdrawal, please see our Privacy Policy.

Reviewer #1: No

Reviewer #2: No

[NOTE: If reviewer comments were submitted as an attachment file, they will be attached to this email and accessible via the submission site. Please log into your account, locate the manuscript record, and check for the action link "View Attachments". If this link does not appear, there are no attachment files to be viewed.]

While revising your submission, please upload your figure files to the Preflight Analysis and Conversion Engine (PACE) digital diagnostic tool, https://pacev2.apexcovantage.com/. PACE helps ensure that figures meet PLOS requirements. To use PACE, you must first register as a user. Registration is free. Then, login and navigate to the UPLOAD tab, where you will find detailed instructions on how to use the tool. If you encounter any issues or have any questions when using PACE, please email us at figures@plos.org. Please note that Supporting Information files do not need this step.

# Author comment: Author response to Decision Letter 0

**DOI:** 10.1371/journal.pone.0235288.r002

5 May 2020

Dear academic reviewers,

Thank you very much for reviewing our manuscript – your suggestions were very helpful!

Please find below our responses to each point raised in your review. We remain ready to address any additional questions or suggestions to improve our manuscript.

Reviewer #1:

Many thanks for your suggestions! We have addressed each one of your comments to improve this manuscript. We agree that there are additional threats that affect C. acutus across its range so we have expanded a description of such in our introduction. We have also updated the bibliography and included the most recent papers that are most relevant to the genetics of the conservation of C. acutus . Where relevant, we have also included papers relevant to new surveys and ecological studies.

1. Author do not consider and include other threats as habitat destruction for many purposes even with more important.

We have added more background in key threats to Crocodylus acutus , incorporated references, and discussed the relevance of genetics in the context of habitat loss and fragmentation. Specifically, we have included a paragraph addressing the consequences of habitat destruction and other contemporary threats that could threaten the species' genetic integrity. These updates can be found in lines 76-85, 545-550, and the References section.

2.An update of bibliography is required.There are updated information about C. acutus surveys and general information and should be included.

To address this comment we have updated our References section to include those papers that better inform our introduction, discussion, and conclusion sections (see list of new references below and the updated References section in the manuscript). If you have any specific suggestions on literature we would still need to add please kindly let us know.

1. UNEP (2020). The Species+ Website. Nairobi, Kenya. Compiled by UNEP-WCMC, Cambridge, UK. Available at: www.speciesplus.net. [Accessed

(04/29/2020)].

2. LeBuff C. Historical review of American crocodiles ( Crocodylus acutus ) along the Florida Gulf Coast. History. 2016;54(3):50–7.

3. IUCN CSG Crocodilian Capacity Building Manual. Available at:

http://www.iucncsg.org/pages/Crocodilian-Capacity-Building-Manual-Home.html.

4. Todesco M, Pascual MA, Owens GL, Ostevik KL, Moyers BT, Hübner S, et al. Hybridization and extinction. Evol Appl. 2016;9(7):892–908.

5. Black J. Assessment of crocodile abundance and seasonal effects of salinity on distribution using both boat based and aerial drone surveys. Purdue University Graduate School; 2019.

6. Cissell JR, Steinberg MK. Human Landscape Modification in Placencia, Stann Creek District, Belize: Possible Implications for Crocodile Hybridization. J Lat Am Geogr. 2020;19(2):218–42.

7. Ortega-León AM, Santos-Morales AH, Zamora-Abrego JG, Pérez-Mendoza HA. Analysis of the population dynamics of the endangered American crocodile, Crocodylus acutus in Paramillo National Natural Park. Mar Freshw Res [Internet]. 2020; Available from: https://doi.org/10.1071/MF19026

8. Machkour-M’Rabet S, Hénaut Y, Charruau P, Gevrey M, Winterton P, Legal L.Between introgression events and fragmentation, islands are the last refuge for the American crocodile in Caribbean Mexico. Mar Biol. 2009;156(6):1321–33.

9. Tellez M, Boucher M. The lessons of history and the future of American Crocodile conservation in Belize. Herpetol Rev. 2018;49(3):492–8.

10. Cox K, Brumund B. Crocodile Smiles: How listed species cope in an age of

urbanization. Nat Resour Environ. 2018;32(3):44–7.

11. Weaver JP, Rodriguez D, Venegas-Anaya M, Cedeño-Vázquez JR, Forstner MRJ, Densmore III LD. Genetic characterization of captive Cuban crocodiles

( Crocodylus rhombifer ) and evidence of hybridization with the American

crocodile ( Crocodylus acutus ). J Exp Zool Part A Ecol Genet Physiol.

2008;309(10):649–60.

12. García-Grajales J, Buenrostro-Silva A. Assessment of human–crocodile conflict in Mexico: patterns, trends and hotspots areas. Mar Freshw Res.

2019;70(5):708–20.

13. Mable BK. Conservation of adaptive potential and functional diversity: integrating old and new approaches. Conserv Genet. 2019;20(1):89–100.

14. Milián-García Y, Castellanos-Labarcena J, Russello MA, Amato G. Mitogenomic

investigation reveals a cryptic lineage of Crocodylus in Cuba. Bull Mar Sci.

2018;94(2):329–43.

15. Fitzgerald LA, Walkup D, Chyn K, Buchholtz E, Angeli N, Parker M. The future for reptiles: advances and challenges in the Anthropocene. Encycl Anthr DellaSala, D, M Goldstein (Eds) Elsevier Sci Ltd, Oxford, UK. 2018;163–74.

16. Somaweera R, Brien ML, Platt SG, Manolis C, Webber BL. Direct and indirect interactions with vegetation shape crocodylian ecology at multiple scales. Freshw Biol. 2019;64(2):257–68.

3. An update of CITES appendices for some countries should be added.

We have updated CITES listings (lines 87-90).

Reviewer #2:

Many thanks for your suggestions on highlighting the relevance of our study to inform conservation! This study will hopefully be useful to inform management plans of C. acutus where translocations and reintroductions could happen and where rapid habitat loss and fragmentation can put distinct populations at risk. We have expanded our discussion and introduction sections to better explain how the genetic integrity of C. acutus could be at risk and how our study could prevent further loss of genetic diversity. Additionally, we have further explained the purpose of the statistical analyses conducted for the mtDNA and microsatellite data.

1.It is better to discuss more how the results would improve the conservation of the American crocodile. 

It is certainly important that our findings can inform conservation. We had added a paragraph in the discussion section (lines 544-550) highlighting the importance of informing future management plans to maintain the genetic integrity of the populations.

2.It would be helpful for the non-specialist reader if the authors provide the

general-purpose and characteristic of each statistical analysis.

To further describe the characteristics and purpose of the statistical analysis conducted in our study, we have added lines (299-300) for the nested AMOVA used in the mitochondrial DNA analysis; and lines (316-317) for the Bayesian clustering analyses used in the microsatellite study. In addition, in lines 289-299, 319-330, 334-336, and 356-361 we explained more in-depth the statistical analyses performed.

Attachment Submitted filename: Response to Reviewers.pdf Click here for additional data file.

# Peer review: Decision Letter 1

**DOI:** 10.1371/journal.pone.0235288.r003

**Authors:** Tzen-Yuh Chiang

12 Jun 2020

High levels of population genetic differentiation in the American crocodile (Crocodylus acutus)

PONE-D-20-06980R1

Dear Dr. Rossi,

We’re pleased to inform you that your manuscript has been judged scientifically suitable for publication and will be formally accepted for publication once it meets all outstanding technical requirements.

Within one week, you’ll receive an e-mail detailing the required amendments. When these have been addressed, you’ll receive a formal acceptance letter and your manuscript will be scheduled for publication.

An invoice for payment will follow shortly after the formal acceptance. To ensure an efficient process, please log into Editorial Manager at http://www.editorialmanager.com/pone/, click the 'Update My Information' link at the top of the page, and double check that your user information is up-to-date. If you have any billing related questions, please contact our Author Billing department directly at authorbilling@plos.org.

If your institution or institutions have a press office, please notify them about your upcoming paper to help maximize its impact. If they’ll be preparing press materials, please inform our press team as soon as possible -- no later than 48 hours after receiving the formal acceptance. Your manuscript will remain under strict press embargo until 2 pm Eastern Time on the date of publication. For more information, please contact onepress@plos.org.

Kind regards,

Tzen-Yuh Chiang

Academic Editor

PLOS ONE

Additional Editor Comments (optional):

Reviewers' comments:

Reviewer's Responses to Questions

**Comments to the Author**

1. If the authors have adequately addressed your comments raised in a previous round of review and you feel that this manuscript is now acceptable for publication, you may indicate that here to bypass the “Comments to the Author” section, enter your conflict of interest statement in the “Confidential to Editor” section, and submit your "Accept" recommendation.

Reviewer #2: All comments have been addressed

**********

2. Is the manuscript technically sound, and do the data support the conclusions?

The manuscript must describe a technically sound piece of scientific research with data that supports the conclusions. Experiments must have been conducted rigorously, with appropriate controls, replication, and sample sizes. The conclusions must be drawn appropriately based on the data presented. 

Reviewer #2: Yes

**********

3. Has the statistical analysis been performed appropriately and rigorously? 

Reviewer #2: Yes

**********

4. Have the authors made all data underlying the findings in their manuscript fully available?

The PLOS Data policy requires authors to make all data underlying the findings described in their manuscript fully available without restriction, with rare exception (please refer to the Data Availability Statement in the manuscript PDF file). The data should be provided as part of the manuscript or its supporting information, or deposited to a public repository. For example, in addition to summary statistics, the data points behind means, medians and variance measures should be available. If there are restrictions on publicly sharing data—e.g. participant privacy or use of data from a third party—those must be specified.

Reviewer #2: Yes

**********

5. Is the manuscript presented in an intelligible fashion and written in standard English?

PLOS ONE does not copyedit accepted manuscripts, so the language in submitted articles must be clear, correct, and unambiguous. Any typographical or grammatical errors should be corrected at revision, so please note any specific errors here.

Reviewer #2: Yes

**********

6. Review Comments to the Author

Please use the space provided to explain your answers to the questions above. You may also include additional comments for the author, including concerns about dual publication, research ethics, or publication ethics. (Please upload your review as an attachment if it exceeds 20,000 characters)

Reviewer #2: The authors addressed all of my concerns. It is a fascinating manuscript, although it is still not crystal clear how the authors would apply this information for the conservation purpose. However, I don’t have any additional concerns about the publication.

**********

7. PLOS authors have the option to publish the peer review history of their article (what does this mean?). If published, this will include your full peer review and any attached files.

If you choose “no”, your identity will remain anonymous but your review may still be made public.

**Do you want your identity to be public for this peer review?** For information about this choice, including consent withdrawal, please see our Privacy Policy.

Reviewer #2: No
